# A discrete element simulation study of paver screed and hot mix asphalt interaction

**DOI:** 10.1038/s41598-025-98928-7

**Published:** 2025-05-07

**Authors:** Leandro Harries, Stefan Böhm, Jia Liu

**Affiliations:** Institute of Transportation Infrastructure Engineering, Technical University of Darmstadt, Darmstadt, Germany

**Keywords:** Asphalt, Paving, Compaction, Simulation, DEM, Discrete element modelling, Paver, Screed, Civil engineering, Mechanical properties

## Abstract

Paving hot mix asphalt (HMA) using an asphalt paver is a complex process requiring precise adjustments of compaction units such as the tamper and vibration unit to suit varying conditions. Despite decades of advancements, the process remains largely dependent on operator experience, and scientifically validated guidelines for optimizing key parameters like tamper and vibration frequency, as well as paving speed, are lacking. Field tests are costly and limited in scope, while internal material movements during compaction are difficult to observe. This study addresses these challenges by employing a full-scale simulation using a calibrated Discrete Element Method (DEM) model to analyze HMA’s flow and pre-compaction behavior. Key findings include insights into the flow and distribution of asphalt material within the auger tunnel and under the screed. The study reveals that the tamper’s compaction force is primarily directed toward the chamfer and has a significant depth effect, achieving considerable pre-compaction of the material. The paving process generates distinct force distributions and particle rearrangements, with key zones of high wear impact identified, especially on the tamper chamfer. Pre-compaction due to the screed’s dead weight is limited, highlighting the tamper’s critical role in achieving optimal compaction. These insights suggest improvements for future paver design, including potential modifications to the screed’s geometry and the introduction of heating elements on the screed front wall to reduce the risk of material cooling and ensure more homogeneous paving. This research contributes to enhancing the understanding of machine-material interactions during asphalt paving, offering practical recommendations for improving paving efficiency and longevity of road surfaces.

## Introduction and background

Ensuring asphalt pavement durability requires sufficient deformation resistance and effective load transfer to the subgrade. Proper compaction is essential, as inadequate paving and compaction accelerate cracking, rutting, and grain disintegration^1^. Errors in paver settings can reduce pavement lifespan and increase both costs and environmental impact. The compaction process occurs in two stages, initial compaction by the screed and final compaction by rollers, where the initial phase is particularly critical, as later corrections are limited.

**Fig. 1:**
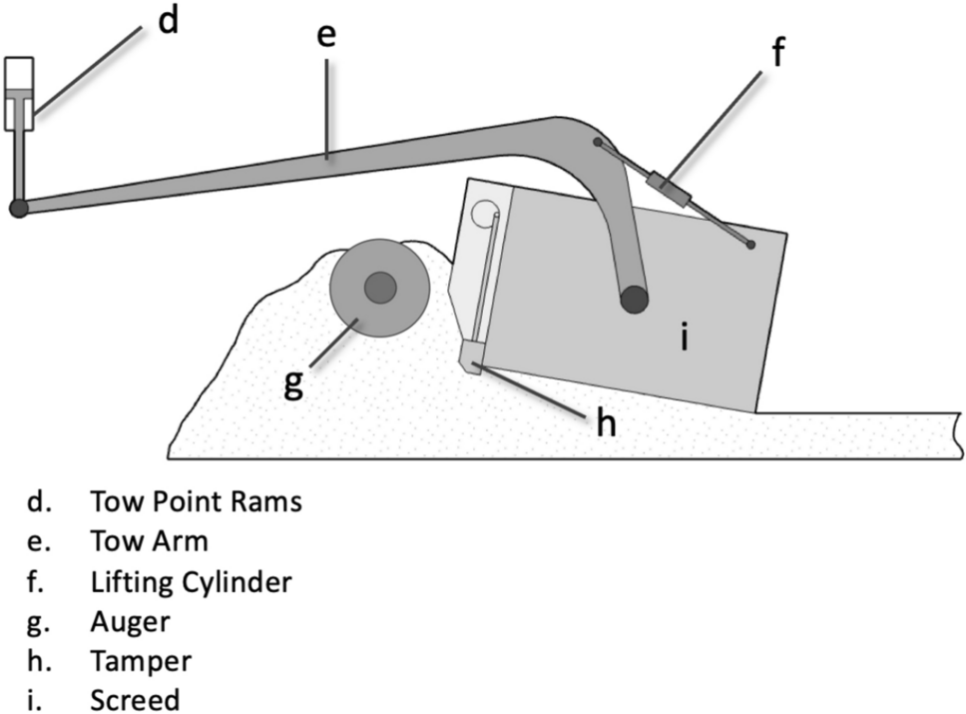
Cross section of paver screed (figure created with Apache Open Office^3^ version 4.1.10; https://www.openoffice.org).

Asphalt pavers are widely used for road construction and perform a complex paving process involving material reception, distribution, and pre-compaction. The screed, functioning as a “floating” unit pulled by two tow arms, provides pre-compaction through its own weight and, in many cases, additional compaction units such as tampers and vibratory systems. Figure 1 illustrates a cross-section of a typical paver screed, highlighting the arrangement of its compaction components. Adjusting these systems appropriately is essential, yet challenging, given the influence of external factors such as mix type, temperature, and desired layer thickness. Despite decades of research, the current paving process remains highly dependent on operator experience and lacks scientifically validated guidelines for optimizing key parameters such as tamper frequency, vibration frequency, and paving speed^2^.

Numerous studies have attempted to understand the mechanisms between paver screeds and hot mix asphalt (HMA). Early lab experiments by Böhmer^4^ demonstrated that increasing tamper and vibration frequencies can enhance compaction but may also cause grain fragmentation at excessively high values. Renner and Schaetz^5^ further examined friction dynamics between the screed plate and HMA. Utterodt^2^ conducted field tests to correlate material behavior with tow arm forces and showed that screed penetration increases with hotter mixes due to reduced viscosity. Jia et al.^6^ and Wan and Jia^7^ developed dynamic and rheological models to simulate these interactions, revealing complex relationships between excitation frequencies and compaction efficiency, including resonant peaks that contradict earlier linear assumptions. Wang et al.^8^ applied Discrete Element Method (DEM) simulations to model paving, albeit with simplifications that neglected the floating behavior of the screed.

While prior studies provide valuable insights, challenges remain in visualizing and experimentally evaluating the internal compaction mechanisms during screed operation. Field trials are resource-intensive and limited in scope, while key interactions beneath the screed remain hidden from observation. Therefore, this study aims to deepen the understanding of the pre-compaction phase through a coupled Discrete Element Method (DEM) and Multi Body Dynamics (MBD) approach. By simulating the complex interaction between paver screeds and hot mix asphalt, the research seeks to identify optimal operating parameters and ultimately contribute to longer-lasting pavement structures with lower maintenance costs.

## Methodology and processing

**Fig. 2:**
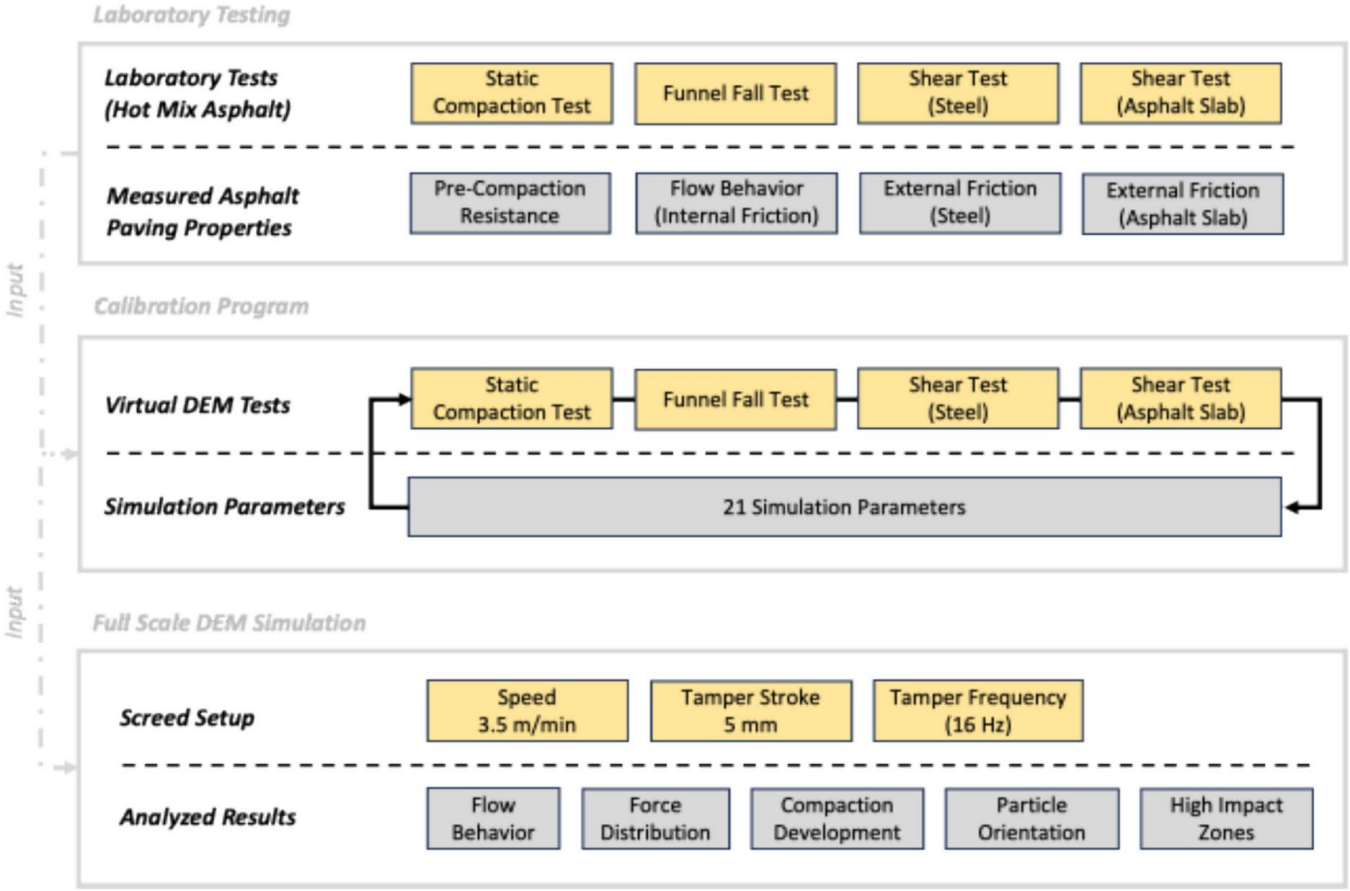
Overview flow charts of the applied methods.

The full-scale simulation of the paver screed requires determining and analyzing the paving properties of the HMA according to^9,10^ through laboratory testing. This data is then used as input for the calibration program according to^11^. Figure 2 provides an overview.

### Calibration program

A total of 21 simulation parameters must be determined when carrying out the relatively complex calibration program. The Edinburgh Elasto-Plastic Adhesion (EEPA) contact model, which is also recommended by Müller^12^ for the simulation of HMA, was used to describe the contact mechanics between the asphalt particles. The contact mechanics between the asphalt particles and the machine parts is described by a simple Hertz-Mindlin model, as proposed for example in^8^. The grading curve of the asphalt mix is represented by a simplified two-particle size mixture and the particle shape consists of a two-sphere clump. This simplified representation enables large-scale simulations to be carried out with economic simulation times. As shown in detail and validated by the calibration program in^11^, the relevant asphalt paving properties can be realistically reproduced despite the assumed simplifications.

**Fig. 3:**
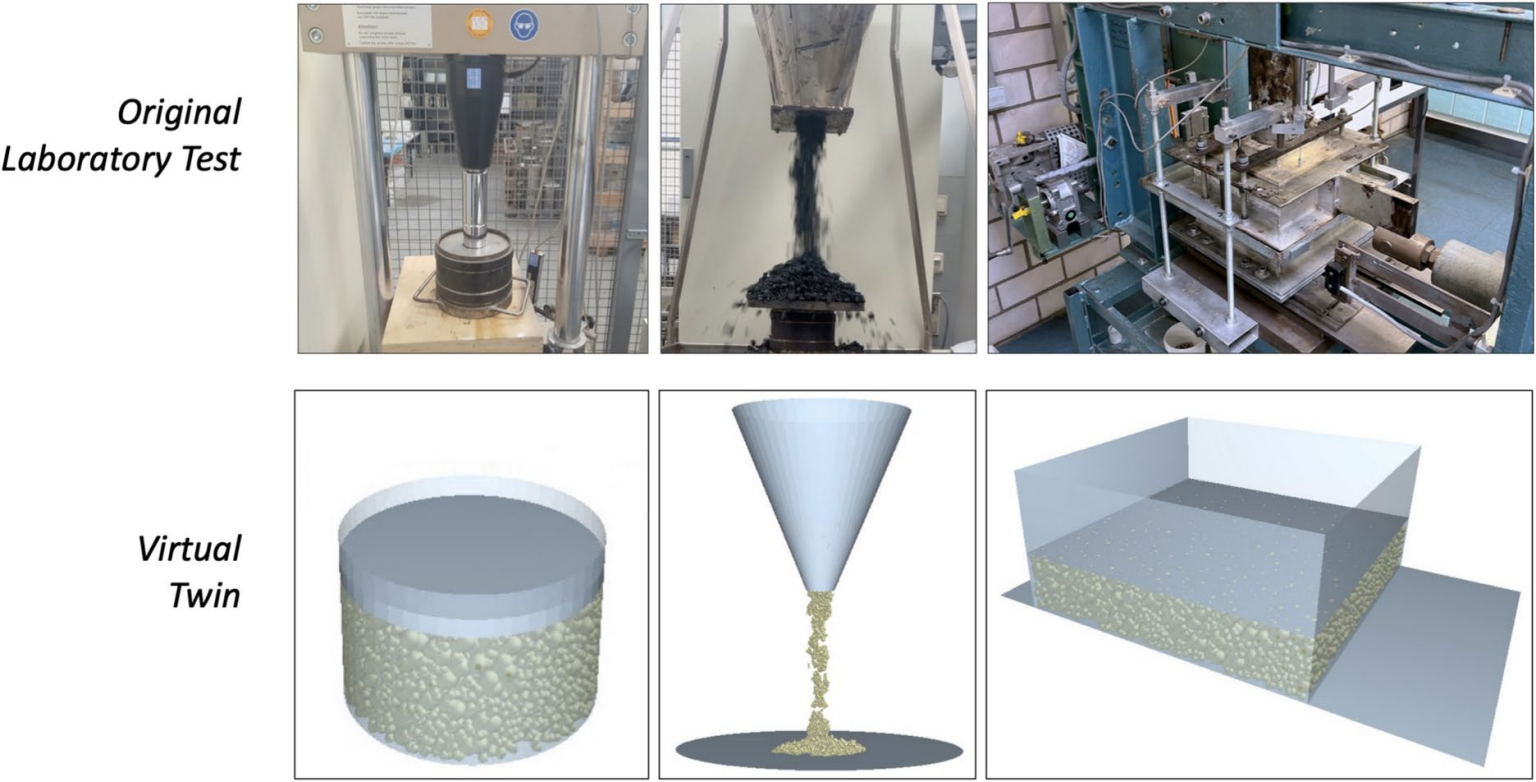
Laboratory setup (from left to right)—static press test, funnel fall test, shear test (3D.images created with Altair EDEM^13^ version 2022.0; https://www.altair.de/edem).

To carry out the calibration program, a total of three laboratory tests—the static press test, the funnel fall test and the shear frame test—must be performed to characterize the paving properties mentioned (cf. Fig. 3). In the second step, the laboratory tests are to be carried out iteratively as virtual twins in order to finally determine the required simulation parameters.

### Full scale simulation

**Fig. 4:**
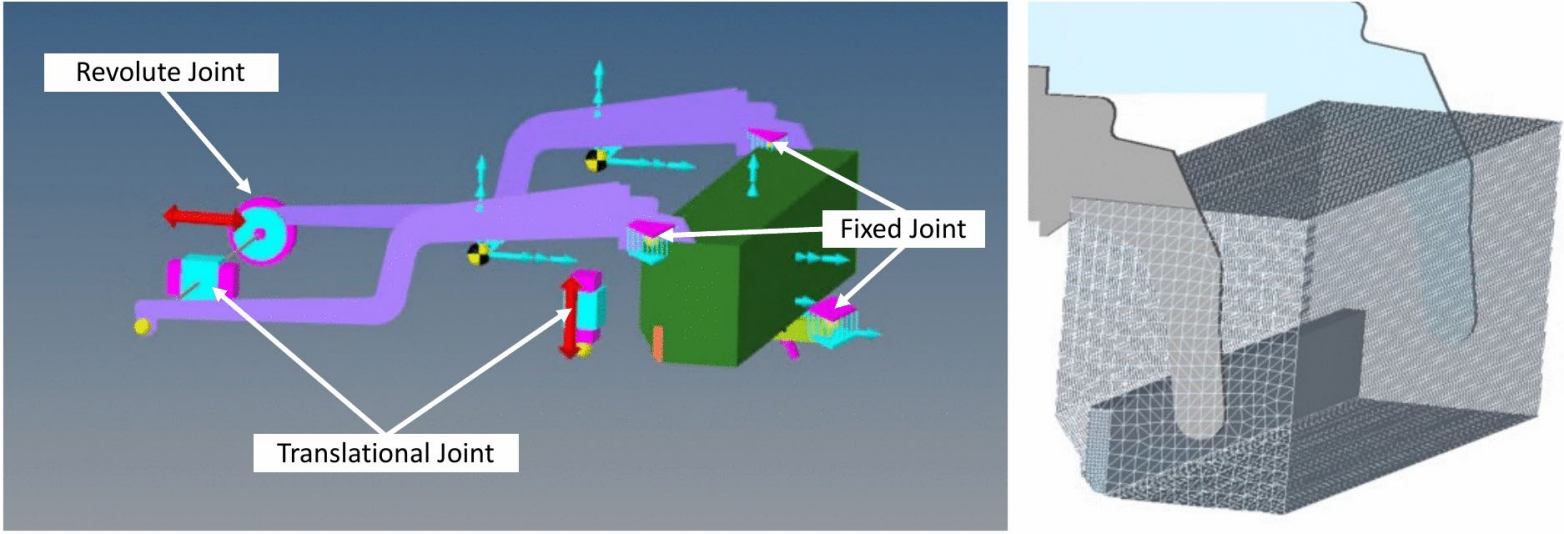
left—Paver screed for simulation with joints (3D.image created with Altair MotionView^15^ version 2021.2; https://www.altair.de/motionsolve/motionview); right—meshed paver screed (3D.image created with Altair HyperMesh^17^ version 2023.0; https://www.altair.de/hypermesh).

For the coupled MDB/DEM full scale simulation, a CAD screed model based on the ABG VB 78 screed was designed using Autodesk Fusion^14^. The 2.5 m wide model was simplified so that only the base screed remained. In addition, small attachments and bolts etc. were removed, as these have no influence on the paving behavior and only lead to an increase in simulation time. Figure 4 (left) shows a part of this model that is relevant for the MBD simulation, consisting of the base screed tow arms and tamper bar, which was imported into MotionView/MotionSolve^15^. The individual components are connected to each other via corresponding joints. As the connection between the tow arms and the screed is rigid, fixed joints were used here. To enable the tamper bar to move vertically, it was connected to the screed via a translational joint. The forward movement of the screed was also ensured by a translational joint. To enable the floating state of the screed, the tow arms were fitted with a revolute joint. The tow point was set to a height of 10 cm.

In order to analyze the wear, using the Relative Wear Model (details in^16^), on the machine side, it is necessary to provide the screed and the tamper with a fine mesh. This mesh was created using the HyperMesh software^17^. The results can be seen in Fig. 4 (right).

**Fig. 5:**
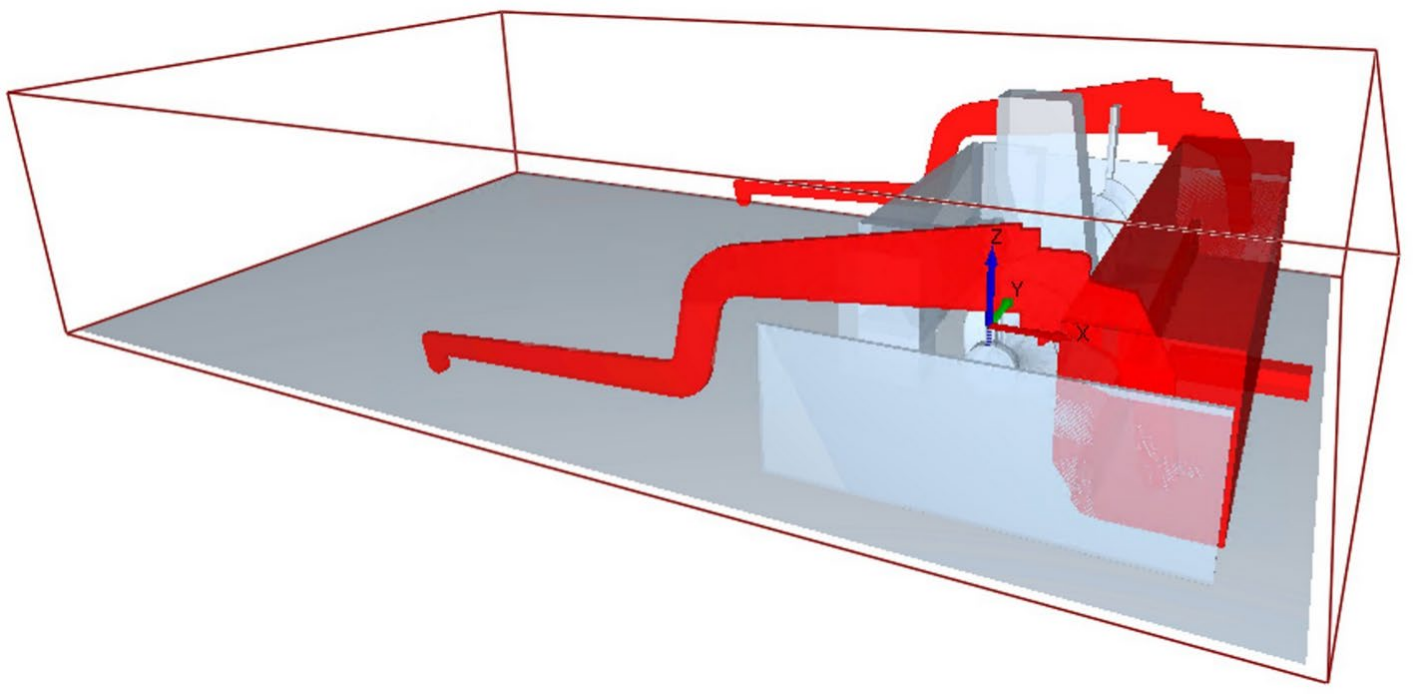
Tow arms and paver screed (3D.image created with Altair EDEM^13^ version 2022.0; https://www.altair.de/edem).

The other components, such as the auger, the auger mounting, the side parts and parts of the conveyor belt were created within the EDEM software, as these are not part of the floating system (cf. Fig. 5).

STEP functions were defined in MotionView^15^ to set the system in motion. These define the forward movement of the screed, which was applied to the horizontal translational joint as well as the sinusoidal vertical movement of the tamper. The paving and tamper speeds in Fig. 2 are based on the recommendations in^18,19^ for paving AC 16 BS. The tamper stroke is determined by the screed type which in this case consistently operates at a fixed 5 mm stroke.

To analyze density increase, bulk density sensors must be placed in front of and beneath the screed. Densities calculated by EDEM, based on the simplified grading curve and particle shape, are significantly lower than actual measured values. Therefore, degrees of compaction are calculated using Eq. (1) for better visualization. The reference value of 2000 kg/m^3^ is derived from a virtual Marshall sample, which approximates this density when produced with the same grading curve and particle shapes.

1 $${k}_{2000} = A\frac{{\rho }_{Sensor}}{2000 \frac{\text{kg}}{{\text{m}}^{3}}}$$

## Simulation results

**Fig. 6:**
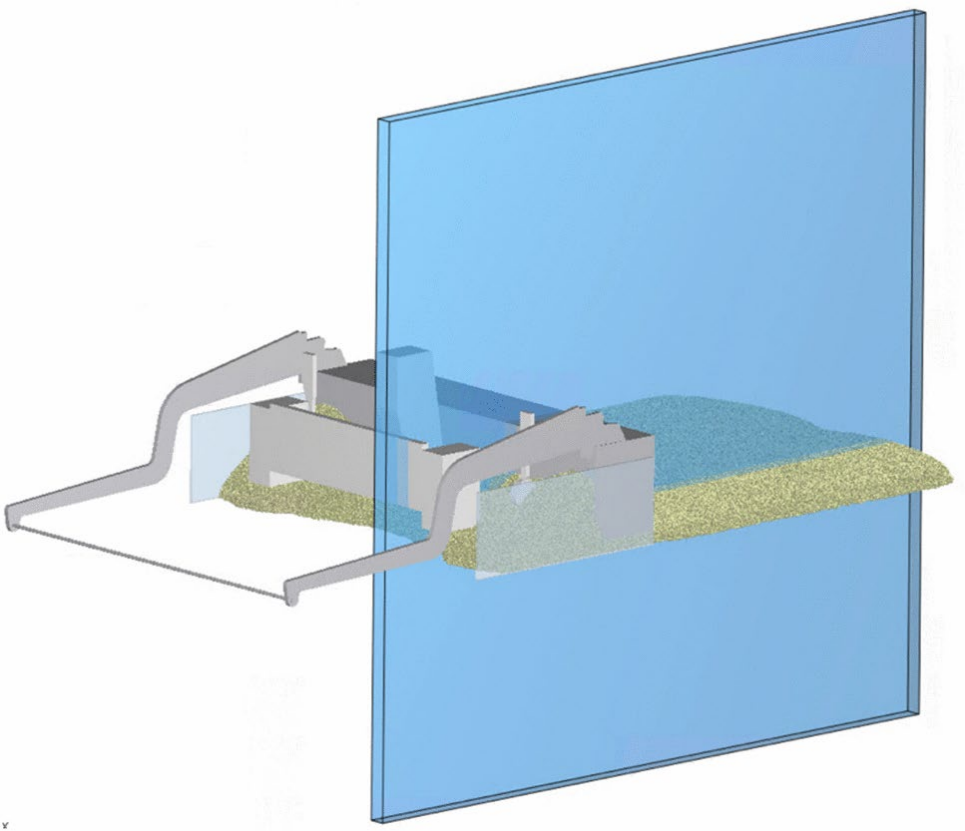
Clipping section for cross section analysis (3D.image created with Altair EDEM^13^ version 2022.0; https://www.altair.de/edem).

The evaluation of the full-scale simulation is carried out mostly qualitatively and includes the analysis of the flow behavior, the force distribution, the grain orientation and the development of the densities during paving with the paver screed. To identify areas of high impact, the machine parts screed and tamper are analyzed using the Relative Wear Model. Although the full-scale simulation was carried out in three dimensions, the evaluation of the results mainly takes place in a cross-section view. Only one cross-section is considered, offset 0.45 m from the screed center with a thickness of 0.2 m (cf. Fig. 6), as preliminary tests showed no significant differences in the evaluated parameters.

### Flow behavior

**Fig. 7:**
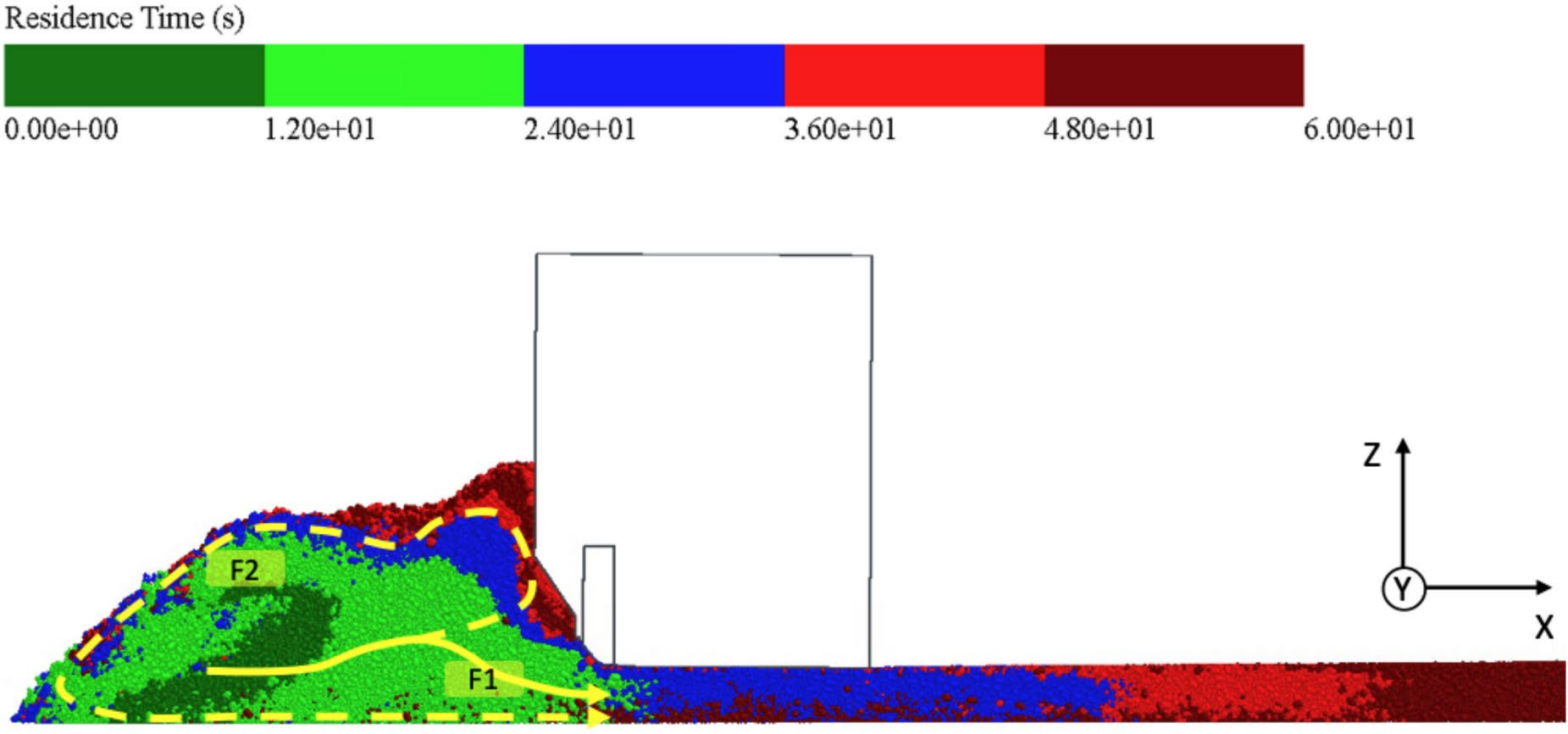
Simulation particles colored according to residence time (3D.image created with Altair EDEM^13^ version 2022.0; https://www.altair.de/edem).

Figure 7 shows a representation of the cross section with simulation particles that are colored according to the residence time in seconds. The residence time describes the duration that the particles have already been present within the simulation. As the particles are continuously added during paving, there are different colorations. The dark green particles have only been part of the simulation for a short time, while the dark red particles were already generated at the start of the simulation. The right part of the figure shows the layer that has already been placed. Since the screed is moving from right to left, the “oldest” particles are consequently at the far-right edge. To analyze the flow behavior, however, the area in front of the screed in the auger tunnel must be considered. It can be noted that the asphalt mix is not only carried outwards along the y-axis by the auger movement, but that particles are also carried upwards along the z-axis (cf. Fig. 7, flow path F2). Another large proportion of the particles remain on the front wall of the screed. This can be clearly recognized by the light and dark red areas directly in front of the screed. Other particles appear to follow path F2 and are transported forwards by the auger movements against the x-direction in the direction of travel until they are finally transported along the front area of the heap towards the base layer. Mixed with “younger” particles from the conveyor belt resp. the particle factory geometry, the particles remain on the base layer until they are paved over by the screed. Over a large area, however, a sort of wedge is formed (cf. Fig. 7, flow path F1), which makes up the main part of the particle transport and favors “younger” particles in particular in that they migrate relatively quickly as soon as they reach the auger tunnel into the lower third of the heap and from there reach the screed without detours. Looking at the area under the screed plate, it can be seen that “older” particles are present especially at the bottom due to the two flow paths described.

### Force distribution

**Fig. 8:**
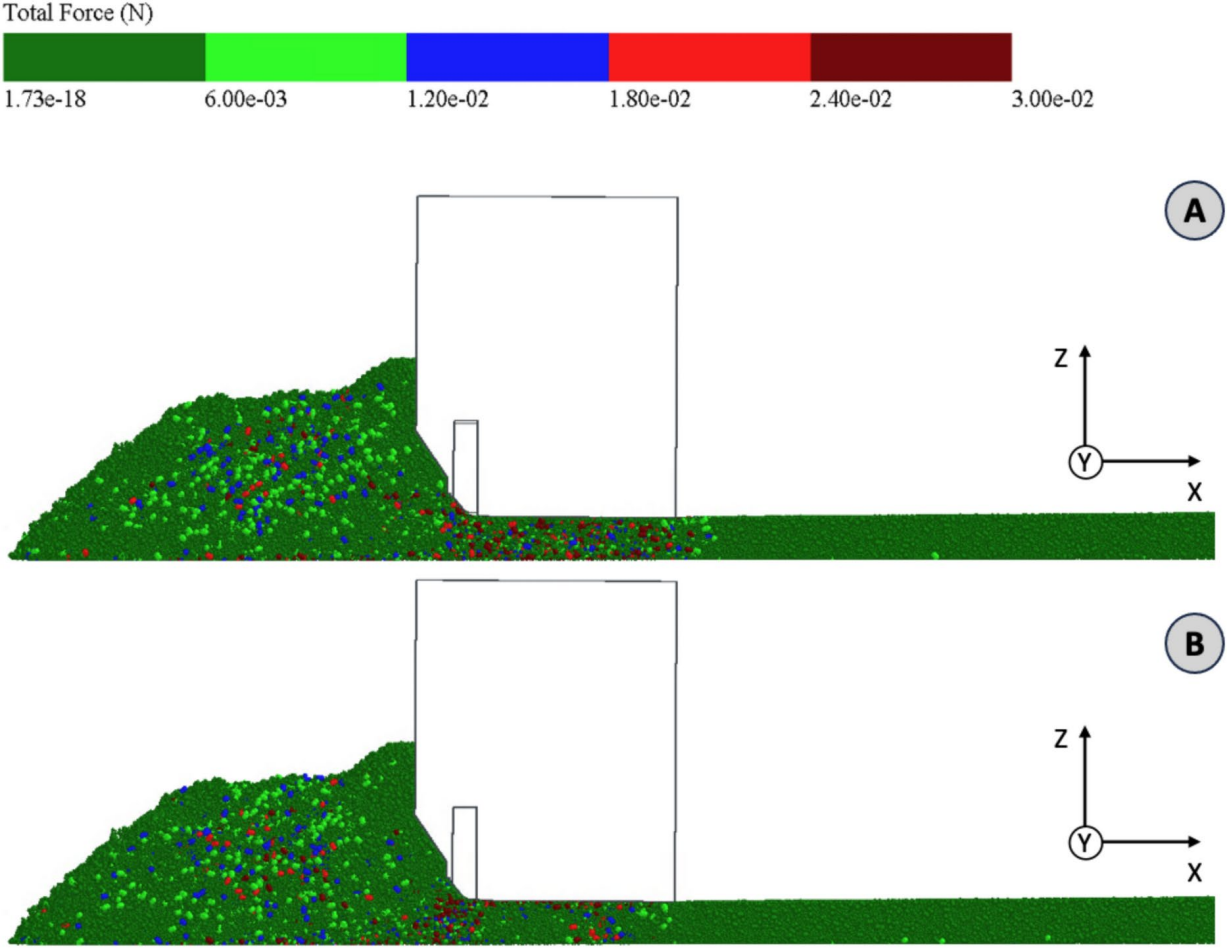
Simulation particles colored according to total force (z-axis) for conditions tamper retracted (**A**) and tamper extended (**B**) (3D.images created with Altair EDEM^13^ version 2022.0; https://www.altair.de/edem).

Figure 8 shows the particles colored in Newtons according to the total vertical force (z-axis) acting on them. The first case considered is when the tamper is fully retracted (A). In the area in front of the screed, a number of forces are acting, which result primarily from the particle flow described above resp. the interaction with the auger. In comparison, the dead weight of the heap has a relatively small influence on the forces acting on the particles in the lower area close to the base layer. There is no accumulation of particles with a strong vertical force effect here. The forward movement of the screed also does not appear to have a particularly high influence on the vertical force acting on the particles on the screed front wall. Considering the area under the screed plate, there is a considerable load acting on the particles. This is distributed fairly homogeneously in both the x-direction and the z-direction. The homogeneous distribution along the z-axis indicates that the screed’s dead weight acts over the entire depth of the layer. However, the homogeneous distribution in the x-direction contradicts the general assumption that the greatest forces occur at the rear edge of the screed due to the principle of the floating screed and the associated moments of force.

If the tamper is fully extended, the distribution of forces results as shown in Fig. 8B. In the area below the screed plate, the vertical forces are significantly reduced. Only in the middle area (x-direction) are isolated particles on which larger forces act. The particles that are in the normal direction to the chamfer of the tamper are most heavily loaded in this phase. The tamper therefore acts predominantly in the direction of the chamfer and not only in the vertical direction. There is also a homogeneous distribution of forces in the z-direction, which indicates a sufficient depth effect of the tamper.

Looking at both paving phases (A) and (B), it can be clearly seen that the force introduced by the tamper stroke leads to a relief in the area of the screed plate visa versa.

### Compaction development

**Fig. 9:**
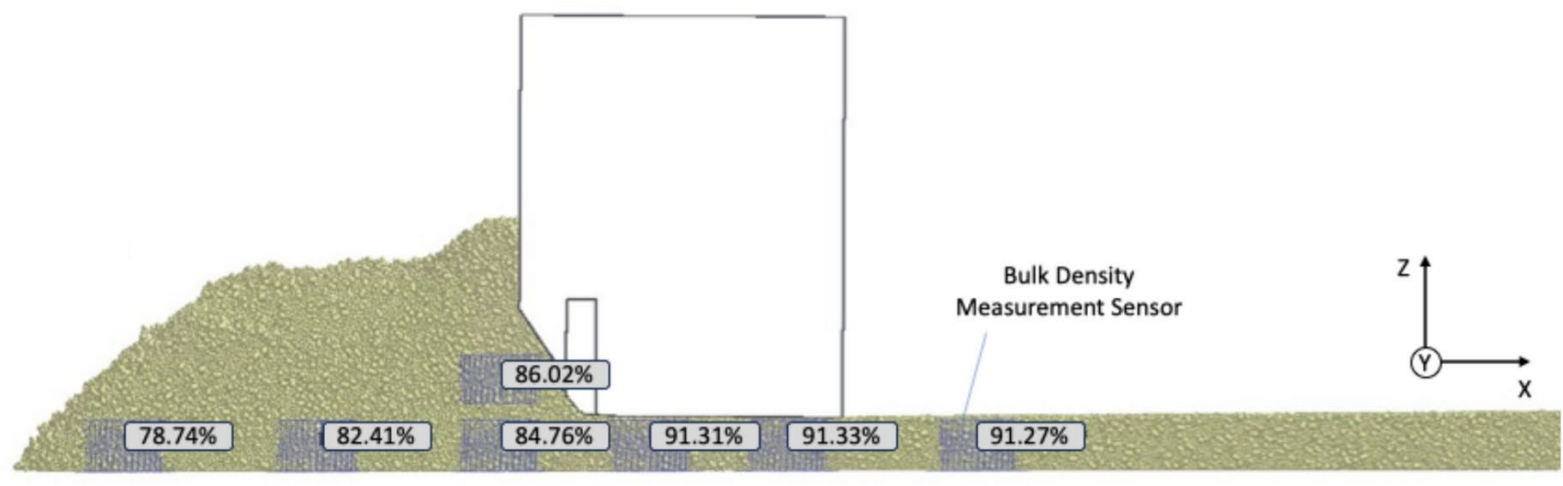
Development of degree of compaction (calculated on basis of a hypothetical reference bulk density) (3D.image created with Altair EDEM^13^ version 2022.0; https://www.altair.de/edem).

In the x-direction, an increase in density is predominantly observed (cf. Fig. 9). For example, the sensor under the auger already has a density that is almost 4% higher than the sensor at the edge of the heap. This is presumably due to the compaction power of the auger, which affects the particles in the center of the heap. A further increase in density can be seen in front of the beveled screed front wall, which in turn is higher than the measured density of the sensor below it. This cannot be explained directly by the normal force acting in the z-direction, but can be explained by the compaction in the x-direction due to the advancement of the screed. Towards the sensor directly behind the tamper, there is again a strong increase in density of almost 7%, which is mainly due to the influence of the tamper. No significant further increase in density can be detected due to the screed’s own weight.

### Particle orientation

**Fig. 10:**
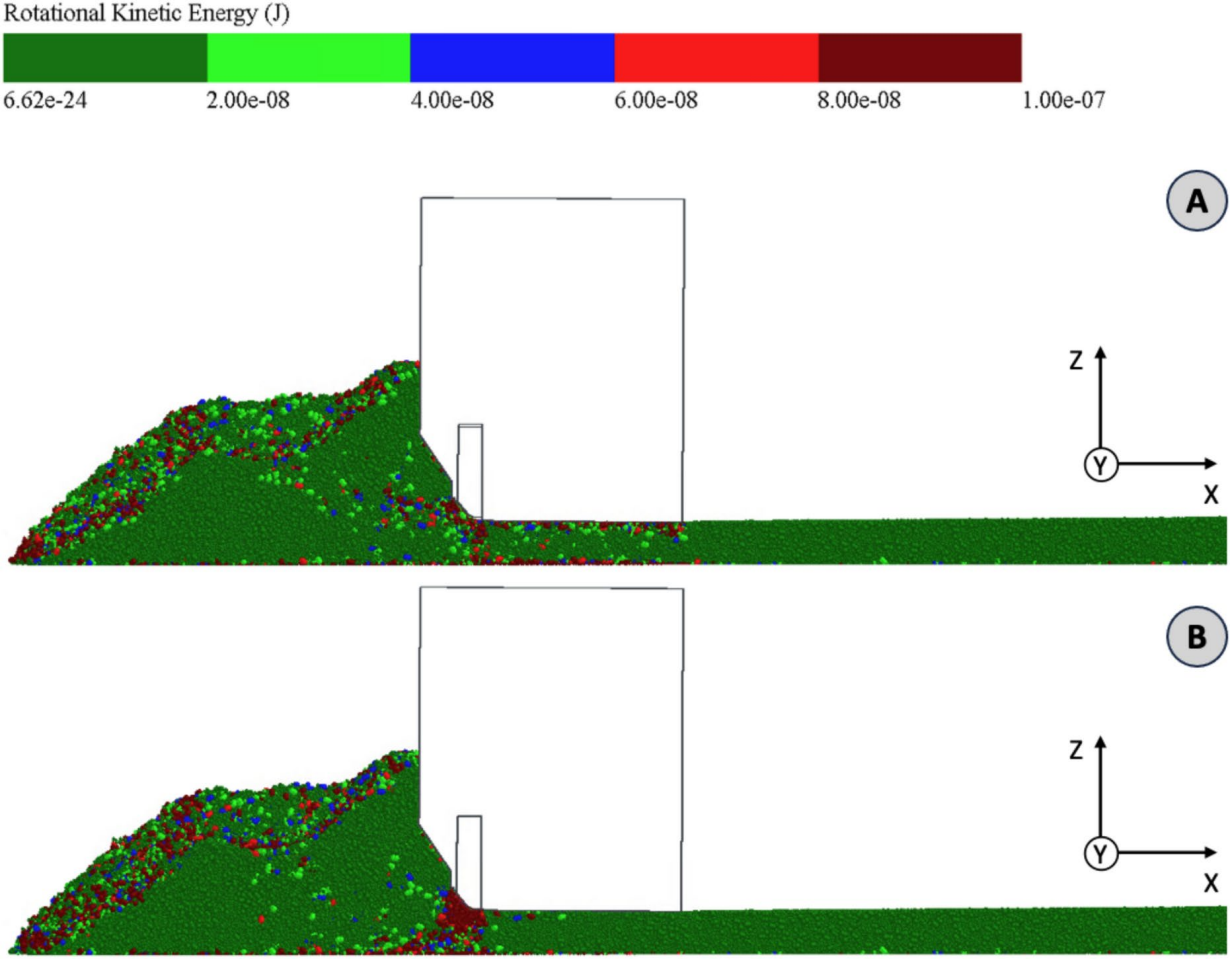
Simulation particles colored according to rotational kinetic energy for conditions tamper retracted (**A**) and tamper extended (**B**) (3D.images created with Altair EDEM^13^ version 2022.0; https://www.altair.de/edem).

In order to analyze how the paving process affects particle orientation and at which point particles orient or align themselves, the rotational kinetic energy in joules is considered. In Fig. 10, the particles are colored according to their rotational kinetic energy. Equivalent to the force analysis, a distinction is also made between the installation state with the tamper retracted (A) and the tamper extended (B).

Condition (A) shows an accumulation of rotational particle movements at the front area of the asphalt heap and the area of the auger blades. This can be explained very well with the derived flow path F2 (cf. Fig. 7). Particle rearrangements also occur along flow path F2. For a large part of the heap, however, relatively little particle rearrangement takes place. This applies in particular to the area under the auger and close to the base layer. In front of the chamfer of the tamper, there is a further accumulation of rotational particle movements, which are initiated by the advance of the screed. Particle rearrangements also take place in the area under the screed at the transition to the screed plate. The reason for this is probably the compaction of the particle structure due to the dead weight of the screed on the one hand and the frictional forces between the screed plate and the particles on the other. Particle rearrangements also take place at the boundary to the base laver in the area under the screed. This is probably also due to the additional compaction and the external frictional forces between the base layer and the particles.

When looking at condition (B), a very similar picture emerges for the heap in front of the screed. There is a clear difference for the area in front of and below the tamper. Due to the tamper stroke, there is a very strong accumulation of rotational particle movements here. Like the forces acting in the z-direction, these are homogeneously distributed over the depth, which again indicates a sufficient depth effect of the tamper. For condition (B), few to no rotational particle movements are recognizable in the area under the screed.

**Fig. 11:**
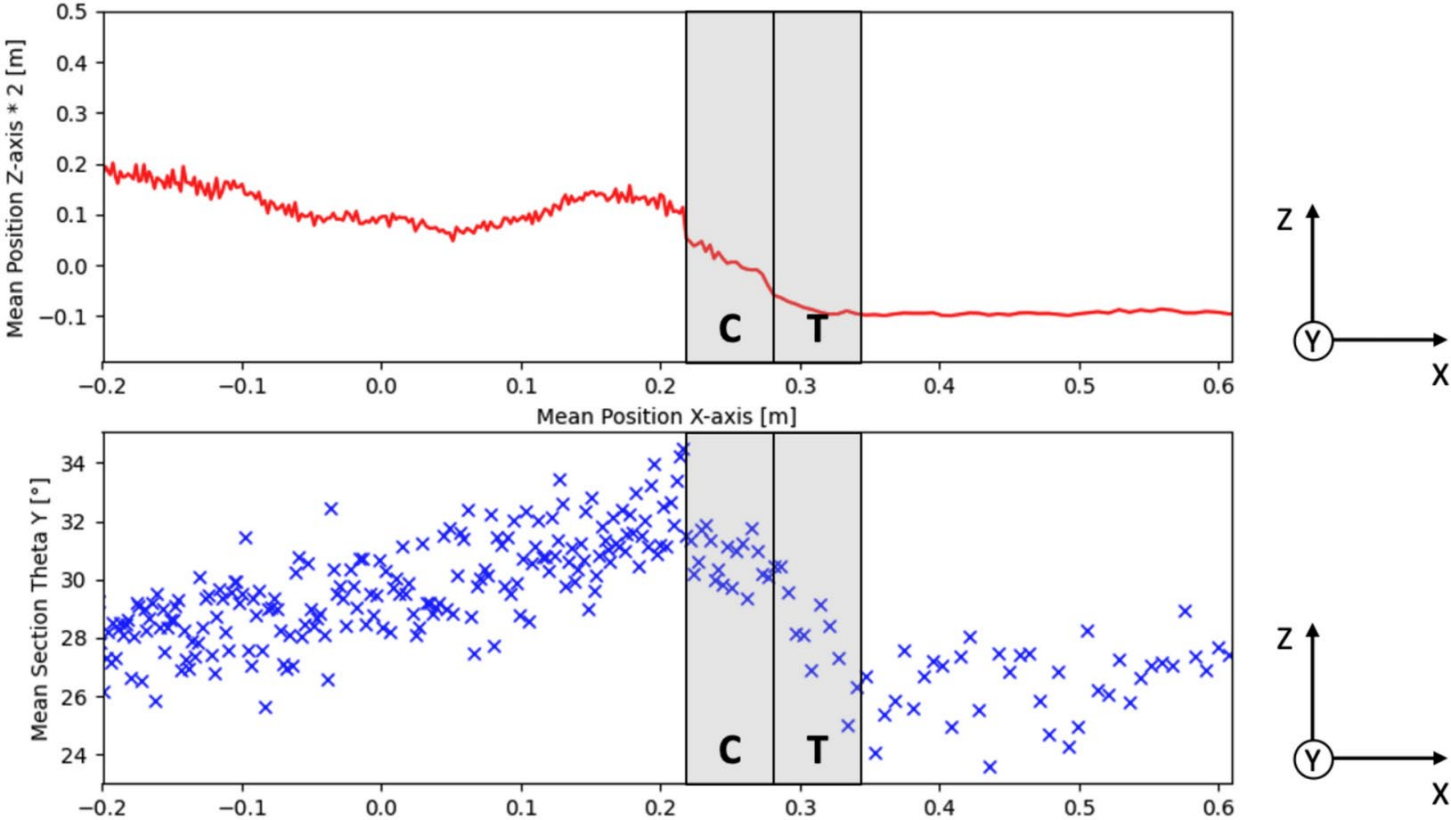
Euler angle (Y-axis) of the particles in area in front of and below screed.

The calculation of the Euler angle around the y-axis allows the actual particle orientation to be observed. The results of the calculation are shown in Fig. 11. In the upper part of the figure, the mean position of the particles in the z-direction is shown twice exaggerated and serves to illustrate the screed geometry. The lower part of the figure shows the mean Euler angles, which were averaged over both the depth (y-axis) and the height (z-axis). They were calculated from rotation matrices using the scipy library^20^. (C) and (T) indicate the beveled area of the screed front wall and the area under the tamper, respectively. A slight increase in the Euler angle up to the screed front wall can be seen in front of the screed. This is probably due to the fact that the particles are pressed against the front wall by the forward movement of the screed and tend to erect as a result. If the particles are located in area (C), they align themselves more horizontally due to the bevel of the front wall. The tamper in area (T) finally leads to a strong horizontal alignment. No further particle rearrangement takes place under the screed plate. These results are therefore consistent with the findings from the consideration of the rotational kinetic energy (cf. Fig. 10).

### Detection of high impact zones

**Fig. 12:**
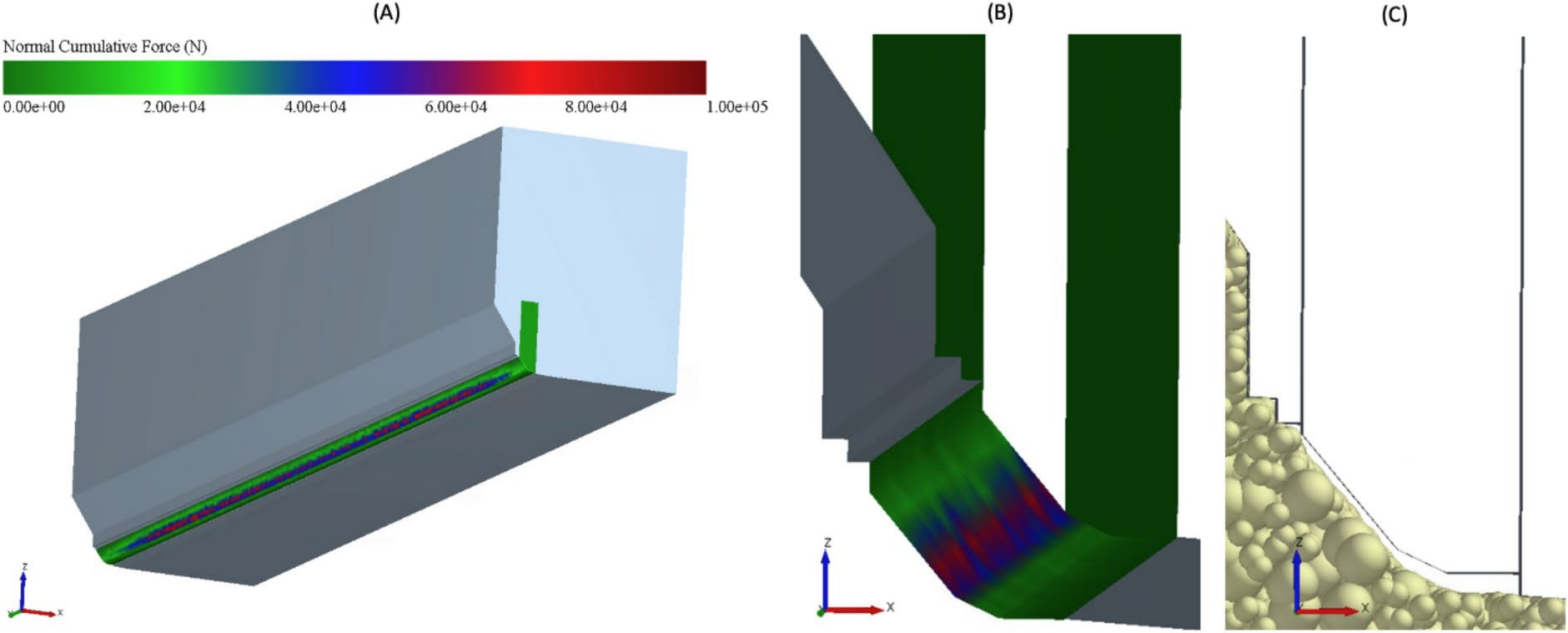
Areas of high impact due to the paving process (tamper) (3D.images created with Altair EDEM^13^ version 2022.0; https://www.altair.de/edem).

Zones of high impact can be identified using the Relative Wear Model or the calculated normal cumulative force. The cumulative normal forces on the tamper resulting from contact with the particles are shown in Fig. 12. The overall view (cf. Fig. 12A) shows higher impact zones in the central area than in the outer area (x-direction) of the tamper bar due to the simulative setup. Contrary to the general understanding and the assumptions in^4^ that the tamper pre-compacts both in the area of the chamfer and with the bottom part, the high impact zones only appear in the area of the upper chamfer (cf. Fig. 12B). The majority of the pre-compaction thus appears to be due to the influence of the chamfer. This can be explained by the results in Fig. 12C. It shows the tamper in a fully retracted state after a compaction cycle. The particles have moved towards the tamper due to the forward movement of the screed and the flow of the particles as a result of the auger movements However, most of the described displacement takes place in the upper chamfer, i.e. in the horizontal x-direction. From below in the z-direction, particles only move upwards to a small extent. In addition, there are elastic components that lead to a re-deformation of the particles but are also relatively small.

## Limitations

Only an HMA calibrated to 160 °C was considered in this study. However, due to the viscosity of the binder, the behavior of HMA is highly temperature dependent. For example, research in^10^ has shown that the pour density increases at higher temperatures and the colder material is less flowable. Field tests in^2^ have shown that, contrary to the common opinion in the literature (e.g. in^18^), lowering the temperature results in a greater layer thickness, which is also explained by the change in flow behavior. In order to extend the applicability of the results to a wider temperature range, further investigations should be carried out with HMA calibrated at other relevant temperature levels.

The paving screed was not varied. However, in the context of practical paving, various paving speeds frequently emerge, contingent on the type of asphalt mix, the paving width and thickness, and numerous additional factors. Of particular note is the effect of the tamper, which exhibits a marked dependence on the paving speed. In^6^, Jia et al. investigated the effect of paving speed on the achievable density of an asphalt mix using a dynamic model based on the theory of vibratory compaction. Their findings revealed a negative correlation between paving speed and the resulting density. To enhance the applicability of the results across a broader speed range, future studies should vary the speed within practical paving speed limits.

The feeding rate was kept constant to maintain a consistent asphalt heap height in the auger tunnel. However, as shown in^10^, the heap height already contributes to increased pre-compaction. Additionally, the authors expect that flow behavior may change, with more particles following the identified flow path F2. Further studies should investigate this effect.

The Relative Wear Model calculates only cumulative normal forces, making it particularly suitable for identifying “high-impact zones”^13^. However, it does not include material-specific parameters like the “wear constant.” To enable quantitative wear rate estimates and assess the long-term impact on paver performance, future studies should incorporate a wear model such as the Archard Wear Model, which accounts for material-specific properties.

In order to be able to carry out the runtime of the DEM simulations economically, the individual particles were represented as two spherical clumps, as was already done e.g. in^21^. This is therefore a relatively rough representation of the particles, which does not allow more precise investigations of the microstructure. However, as the results of the calibration in^11^ have shown, pre-compaction resistance, friction behavior and flow behavior can be realistically reproduced despite the simplified representation.

In this study, the pre-compaction process was only simulated. To validate the results against real-world paver applications, field tests should be conducted and evaluated, focusing on HMA and paver screed interactions.

## Conclusion

To analyze the machine-material interaction, a calibrated full-scale DEM simulation of the paving process with the paver screed was conducted with the calibrated asphalt mix AC 16 BS. The main findings from the simulation can be summarized as follows:

- The investigation of the flow behavior revealed that the HMA forms from the middle of the heap. A significant portion of the asphalt mix moves directly under the screed, while another portion is rotated by the auger and settles in the lower part of the paved layer. Additionally, some asphalt mix accumulates at the upper part directly on the screed front wall where it remains during paving.
- The analysis of the vertical normal force has shown that the compaction effect of the tamper acts primarily in the direction of the chamfer. The results show a pronounced depth effect down to the base layer. A load on the HMA due to the downward movement of the tamper leads to a relief of the HMA under the screed plate.
- The evaluation of the bulk density at several points in front of and under the screed has shown that a pre-compaction of the HMA already occurs due to the dead weight of the asphalt heap (approx. 5%). The tamper stroke is responsible for the main part of the pre-compaction (approx. 7%). Due to the advanced state of compaction, the effect of the screed’s dead weight does not lead to any significant increase in the degree of compaction.
- By considering the kinetic rotational energy and the Euler angle of the particles around the horizontal y-axis, it could be shown that the main part of the rotational particle displacement is caused by the effect of the tamper. The relief of the tamper leads to a small amount of further rotational redistribution at the boundary to the screed plate and the base layer.
- Finally, wear areas of high impact could be identified by investigating the geometry. The high impact areas on the tamper were particularly evident on the chamfer. The horizontal bottom section of the tamper is subjected to comparatively little load, which can be attributed to the flow behavior of the asphalt mix directly in front of and under the tamper.

The analysis of the flow behavior of HMA in the auger tunnel has shown that asphalt mix remains in the upper part directly in front of the screed front wall over longer paving distances. This can lead to cooling of the asphalt mix, which in turn has a negative effect on the paving properties and ultimately leads to inhomogeneities in the paved layer. It is therefore suggested that, in addition to the screed plate and the tamper, the screed front wall should also be heated in order to achieve a temperature level in the auger tunnel that is as homogeneous as possible. This could be particularly important for the future use of temperature-reduced asphalt mixes, as the range of possible paving temperatures is significantly limited. On the other hand, it is suggested that the geometry of the screed front wall be reconsidered. Possibly, a more beveled or concave rounded screed front wall would favor the material flow. It would also be conceivable to cover the screed. In addition to maintaining the paving temperature, this would lead to a reduction in vapor and aerosol emissions in the area of the screed and lead to more effective suction systems.

Investigations of the wear of the tamper by identifying high impact areas have shown that the chamfer of the tamper in particular is heavily loaded because the HMA moves toward the screed front wall, mainly due to the forward movement of the screed. As a result, the tamper only makes a minor contribution to the rearrangement and compaction processes in the lower area. A tamper with a larger beveled or even rounded area would possibly have a stronger effect mechanism. Tamper wear is generally rapid, meaning that the tamper bar has to be replaced once every paving season with moderate to heavy use. This is not only costly, but the increasing wear also leads to a change in the tamper’s operating mechanism, which makes the correct adjustment of the tamper stroke and strokes much more complex. It is therefore recommended to refine the chamfer part of the tamper in particular with an alloy that is as hard as possible.

## Data Availability

Data will be available from the corresponding author upon reasonable request.
